# Practitioner's Bookshelf

**Published:** 1892-05-07

**Authors:** 


### PRACTITIONERS' BOOKSHELF.

### DISSECTION GUIDES.*

This book has been written with the object of enabling the student of anatomy to acquire a maximum knowledge of the practical side of his subject with the expenditure of a minimum amount of time. It is essentially a practical work on methods of dissection, and in no way claims to be a systematic treatise on the subjeoc of anatomy. The chief, and indeed the original, feature of the book consists in the fact that the methods of dissection employed are so arranged that the student is enabled to study the superficial and deep structures simultaneously and conjointly. The author also lays great stress on what he calls the *De fil en aiguille* principle in the dissection of the smaller structures generally. It would be difficult to overestimate the importance and practical applicability of this principle, which is admirably illustrated in the introductory portion of the book, and which we hope will be still further expanded in future editions. The arrangement of the work is excellent, and calls for no special comment. The methods employed in the dissection of the head and neck are especially worthy of menticn, and this section will probably be more appreciated by students than any other in the book. The great experience of the author as a teacher of anatomy has enabled him to come to the assistance of the dissector at exactly those places where help and advice are most needed. It is, however, with regret that in a work of so great excellence we find such a sentence as this: "Parts behind inner ankle," "Timothy doth vex all very nervous people." Blemishes of this sort are fortunately few and far between, and we can strongly recommend the book to students of anatomy, who will find in it innumerable valuable and practical hints and advice which they will do well to follow.

* "Dissection Guides." By Thomas Cooke, F.R.C.S. (London and New York, Messrs. Longmans, Green, and Co., 1891.)

### AIDS TO GENERAL PATHOLOGY.

This little work*, consisting of fifty-eight pages, covers the whole subject of general pathology. The amount of information given is enormous, but we can scarcely believe that a treatise of this sort can be of any real service to the student of general pathology. It might possibly be of use to the senior student as a "synopsis" of the subject after he has read some larger work. Condensation of a subject like general pathology can be easily overdone, and it is certainly overdone in this work. The information given, so far as it goes, is, on the whole, most trustworthy.

* "Aids to General Pathology." By Gilbert A. Bannatyne. (Baillière, Tindall, and Cox. 1892.)

